# B cells in autoimmune hepatitis: bystanders or central players?

**DOI:** 10.1007/s00281-022-00937-5

**Published:** 2022-04-29

**Authors:** Christoph Schultheiß, Silja Steinmann, Ansgar W. Lohse, Mascha Binder

**Affiliations:** 1 Department of Internal Medicine IV, Oncology/Hematology, Martin-Luther-University Halle-Wittenberg, Ernst-Grube-Str. 40, 06120 Halle (Saale), Germany; 2 First Department of Medicine, University Medical Center Hamburg-Eppendorf, Martinistraße 52, 20246 Hamburg, Germany

**Keywords:** Autoimmune hepatitis, B cell, Autoantibody, Antigen presentation, Cytokine, B cell, Depletion therapy

## Abstract

B cells are central for the adaptive immune system to mount successful immune responses not only as antibody producers but also as regulators of cellular immunity. These multifaceted features are also reflected in autoimmunity where autoreactive B cells can fuel disease by production of cytotoxic autoantibodies, presentation of autoantigens to autoreactive T cells, and secretion of cytokines and chemokines that either promote detrimental immune activation or impair regulatory T and B cells. The role of B cells and autoantibodies in autoimmune hepatitis (AIH) have been controversially discussed, with typical autoantibodies and hypergammaglobulinemia indicating a key role, while strong HLA class II association suggests T cells as key players. In this review, we summarize current knowledge on B cells in AIH and how different B cell subpopulations may drive AIH progression beyond autoantibodies. We also discuss recent findings of B cell-directed therapies in AIH.

## Introduction

Autoimmune hepatitis (AIH) is a severe chronic and relapsing inflammatory liver disease with a female preponderance characterized by an ongoing autoimmune reaction directed against hepatic autoantigens [1–3]. Like in other autoimmune diseases, the exact pathogenesis remains uncertain. Both B cell and T cell-mediated autoimmunity and immune dysregulation have been proposed as key mechanisms. While the strong association with distinct *HLA-DRB1* alleles strongly supports an important role for CD4^+^ cells in disease development, the characteristic and specific elevation of immunoglobulin G (IgG) levels and the development of both specific and non-specific autoantibodies, which is also of important diagnostic value, support the role of B cells in AIH pathogenesis [1, 3–5]. Furthermore, recently, the establishment of new mouse models and the apparent success of B cell depletion therapies in distinct patient subsets provided further support for the concept of B cells as substantial contributors to AIH immunopathogenesis.

Therefore, in this review, we summarize current knowledge on B cells in AIH and how different B cell subpopulations may drive AIH progression beyond autoantibodies. We also discuss recent findings of B cell depletion in clinical trials as well as further B cell-directed therapeutic approaches beyond depletion.

## B cell development and maturation

**Fig. 1:**
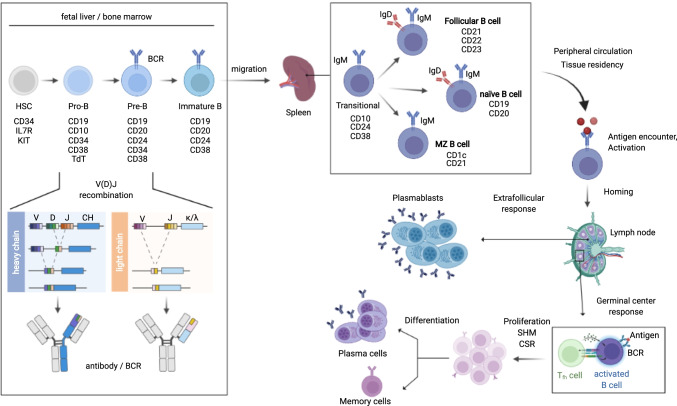
B cell development and differentiation. B cell development is a multistep process initiated in hematopoietic stem cells (HSCs) in the bone marrow or fetal liver. During this process, the B cell receptor (BCR), which consists of a heavy and a light chain, is generated in a complex genomic rearrangement event. This rearrangement, termed V(D)J recombination, randomly assembles one of 40 V, 23 D, and 6 J genes with a constant part (CH) in case of the heavy chains. The light chains lack D chains and can have either a λ or κ constant region. Immature B cells with correctly assembled BCRs finalize their maturation after migration to the spleen, where they either differentiate into naïve, follicular, or marginal zone (MZ) B cells. All stages of B cell development are characterized by sets of surface markers from which a selection is depicted. Upon antigen encounter, activated B cells can either rapidly expand in an extrafollicular response into short-lived plasmablast or engage in a germinal center reaction in secondary lymphoid tissues like lymph nodes. The germinal center reaction is a T cell-assisted BCR diversification process, which facilitates class-switch recombination (CSR) and increases BCR affinity via introduction of random mutations (somatic hypermutation, SHM). Germinal center B cells then differentiate into antibody-secreting plasma cells which can become long-lived plasma (LLP) or memory B cells

B cells constitute one of the essential arms of the adaptive immune system [6]. They are produced from hematopoietic precursor cells throughout life starting in the fetal liver to the bone marrow (BM) in adults (Fig. 1) [7]. During a multistep developmental and selection process, their unique characterizing feature, the B cell receptor (BCR), is generated randomly for each single B cell in a complex genomic rearrangement event generating a diverse B cell repertoire with virtually unlimited specificities [8]. B cells with correctly assembled BCRs that pass checkpoints of central tolerance exit the BM as IgM^+^ immature or transitional B cells and migrate via the bloodstream to the spleen where they complete their maturation process by differentiating into either naïve, follicular, or marginal zone (MZ) B cells after passing peripheral tolerance checkpoints [9, 10]. Based on phenotypic and topographic features, these mature B cells can now circulate in the blood and lymph vessels or populate the secondary lymphoid organs (spleen, lymph nodes, tonsils, and Peyer’s patches) ready for detection of antigens.

Mature B cells which encounter cognate antigen and receive additional activation signals from co-stimulatory molecules expand and differentiate either into short-lived plasmablasts or into germinal center (GC) B cells [11]. While plasmablasts rapidly produce and secrete antibodies corresponding to germline-encoded BCR configurations (naïve BCRs), GC B cells engage in the GC reaction. The GC reaction is a T cell-assisted BCR diversification and selection process which increases affinity towards the initial antigen by introducing random mutations into the paratope sequence during clonal expansion [12, 13]. The GC reaction may also facilitate class-switch recombination to further diversify antibody effector functions. B cells that pass the GC reaction become antibody-secreting plasma cells which can differentiate into long-lived plasma (LLP) or memory B cells providing lasting protection against reinfection.

## Pathogenic B cells in autoimmune diseases

While B cell antigenic selection and maturation is vital to protect against pathogens, it can have detrimental effects when triggered by self-antigens. This may result in autoreactive B cell populations that target an individual’s own tissues or instruct other cells of the adaptive and innate immune system to do so. B cell dyscrasias driven by autoantigen are well defined for some autoimmune diseases [14]. For example, in diabetes, autoantibodies can target insulin producing β-cells or insulin itself [15, 16], in dilated cardiomyopathy (DCM), which is the leading cause for heart failure and heart transplantation in younger adults, circulating autoantibodies mediate organ-specific tissue damage by targeting different epitopes on cardiac myocytes [17, 18]. In pemphigus vulgaris, IgG autoantibodies directed against desmosomes of keratinocytes cause epithelial acantholysis [19]. The pathogenic potential of several autoantibody classes found in rheumatoid arthritis (RA), especially antibodies targeting post-translational modifications like citrullination (ACPA) and carbamylation (anti-CarP antibodies), was recently substantiated [20, 21]. Another prototypic example for the pathogenic role of B cells is systemic lupus erythematosus (SLE), where ISG15-secreting plasmablast expansions are a hallmark of activity [22] and dysregulated GC reactions mediate the positive selection of high-affinity autoantibodies driving pathogenesis [23]. Notably, SLE also shows ongoing somatic hypermutation (SHM) in extrafollicular responses resulting in affinity matured autoantibodies, a feature also found in other autoimmune settings like RA and Sjögren’s syndrome [24]. While these examples illustrate the direct pathogenic potential of autoreactive B cells in different autoimmune diseases, their role in AIH pathogenesis is much less defined.

## The role of B cells as diagnostic markers in AIH

Clinical presentation of AIH is highly variable, ranging from mild and intermittent elevation of liver enzymes to acute and fulminant hepatitis [25]. Since AIH lacks a distinct pathognomonic feature and its etiology is largely unknown, it is diagnosed by exclusion based on clinical, serological, and histopathological features [26–29]. Key to the diagnostic workup are AIH-specific autoantibodies; selective elevation of polyclonal IgG (hyper-IgG), usually in the absence of an elevation of IgA and IgM; and abnormalities in liver histology.

### B cells in AIH liver histology

Liver histology in active AIH is typically characterized by a lympho-plasmacellular infiltrate of the hepatic tissue mediating tissue damage and hepatic necroinflammation. However, the changes in AIH histopathology lack pathognomonic characteristics, as histomorphological changes in chronic AIH mimic findings in chronic viral hepatitis [30–32]. Chronic AIH typically shows portal-based lympho-plasmacellular infiltrates with interface hepatitis (formerly called piece-meal-necrosis) [28, 29]. In the inflammatory infiltrate, plasma cells are detectable in two out of three cases; however, their abundance may vary [30, 33, 34]. Histopathological features such as rosette formation and emperipolesis were described to be typical characteristics of AIH. Recently, these histological findings were considered not to be specific for AIH, but rather reflect the hepatic tissue damage and can act as markers of disease severity regardless of the underling disease [30, 32, 35]. However, predominance of plasma cells as well as appearance in clusters (defined as > 5 plasma cells/one focus) in the context of interface hepatitis were reported to be rather specific for AIH [32]. Thus, despite not being necessarily required for diagnosis, plasma cells can be considered typical in AIH [32]. In immunohistochemistry, expression of CD38 or multiple myeloma-1 (MUM-1) can be used to identify plasma cells and determine their distribution and frequency in hepatic tissue [36].

### Autoantibodies in AIH

Seropositivity for distinct autoantibody classes represents a key diagnostic feature of AIH. In addition, autoantibodies can serve as biomarkers for grouping different AIH subsets. After exclusion of other autoimmune diseases, ANA and SMA (including anti-F-actin antibody) define AIH type 1. Anti-LKM1 and anti-LC2 are characteristic for the AIH type 2 subset. Antibodies to soluble liver antigen/liver pancreas antigen (anti-SLA/LP) are also regarded by many authors as classifying a third subgroup of AIH, AIH type 3. Further autoantibodies such as pANCA, anti-LM, and anti-ASGPR antibodies have also been discussed as characteristic markers of AIH (Table 1).

**Table 1:** Autoantibodies in AIH

Subtype	Antibodies	Target structures	frequency in AIH patients	Clinical features	Presence in concurrent diseases	
					Hepatic	Extrahepatic
AIH type 1	ANA [29]	Chromatin Histones Centromere ds- and ss-DNA Cyclin A Ribonucleoproteins	50–70%		PBC PSC NAFLD DILI HBV HCV	Healthy individuals SLE RA Scleroderma Sjögren’s Syndrome
	SMA [205] (F-Actin)	(Filamentous) actin, tubulin, or intermediate filaments	50%	Correlate with inflammatory activity in adult patients with AIH		
AIH type 2	Anti-LKM-1 [1, 205, 206]	CYP 2D6	Pediatric AIH-patients: up to 20–30% Adult AIH-patients: up to 10%	Early onset of disease More aggressive disease course	HCV	
	Anti-LKM-3 [207]	UGTs	19% of AIH type 2		HCV HDV	
	Anti-LC1 [208]	FTCD (formiminotransferase cyclodeaminase)	30% of AIH type 2		HCV	
AIH type 3	Anti-SLA/LP [209–212]	SepSecS	10–20%	Potentially more aggressive disease course Worse disease outcome		
Further antibodies						
	Anti-ASGPR [213]	Anti-ASGPR	24–82%	Levels might correlate with disease activity [213]	PBC HBV HCV	
	p-ANCA/p-ANNA [214–216]	Unclear, tubulin-beta chain?	AIH-1: 65–96% AIH-2: 13%		PSC HBV HCV	IBD Microscopic polyangiitis Eosinophilic granulomatosis with polyangiitis
Seronegative [134, 217]			10–15%	Acute presentation Often development of autoantibodies upon follow-up		healthy individuals

Abbreviations: *AIH* autoimmune hepatitis, *DILI* drug-induced liver injury, *HBV* viral hepatitis B, *HCV* viral hepatitis C, *HDV* viral hepatitis D, *IBD* inflammatory bowel disease, *NAFLD* non-alcoholic fatty liver disease, *PBC* primary biliary cholangitis, *PSC* primary sclerosing cholangitis, *SLE* systemic lupus erythematosus, *RA* rheumatoid arthritis

Only very few patients lack any type of autoantibody. Autoantibody negativity may, however, occur at acute presentation, but most of these cases develop seropositivity upon further follow-up [37]. While anti-SLA/LP antibodies are highly specific for autoimmune hepatitis, ANA, SMA, and to a lesser extent anti-LKM1 can also be found in other liver diseases or even other autoimmune diseases [38]. Hepatotropic and non-hepatotropic viral infections can lead to a transient development and/or increase of these autoantibodies [38–41]. This limited degree of specificity thus calls into question a direct pathogenic role of autoantibodies in AIH. On the other hand, a high degree of specificity, as observed for anti-SLA/LP, including a high degree of epitope specificity, speaks strongly in favor of a pathogenic role [42, 43]. Albeit a single case report, transfer of maternal SLA/LP autoantibodies both in utero and via breast feeding did not lead to any hepatitis in the newborn [44].

Since these conventional autoantibodies lack diagnostic sensitivity and accuracy [45], several groups aimed to identify other potential targets for more precise diagnostic differentiation of AIH from other related diseases [46–49]. While these efforts identified more than 80 potential targets, only one recent publication systematically validated their findings [50]. Taubert et al. report that polyreactive IgG against HIP1R/BSA proved to be more specific than ANA, anti-SMA, anti-LKM1, anti-SLA/LP, and other autoantibodies [50]. HIP1R/BSA reactivity was detected in up to 88% of otherwise seronegative patients and in up to 71% of AIH patients with normal IgG levels [50]. Another class of non-conventional autoantibodies are programmed cell death 1 (PD-1)-targeting antibodies found in type 1 AIH where they correlated with levels of bilirubin and alanine aminotransferase but not with IgG [51]. PD-1 and its ligands PD-L1 and PD-L2 constitute a co-inhibitory signaling axis that limits lymphocyte activation and is key for central and peripheral tolerance [52–54]. This essential function is illustrated by several PD-1 knockout mouse models that develop a broad spectrum of autoimmune diseases like arthritis, lupus-like glomerulonephritis, diabetes, or fatal dilated cardiomyopathy [54]. In the cancer context, antibody-mediated blockade of the PD-1/PD-L1 axis helps to overcome cancer immune escape on the other hand favoring adverse autoimmune events including AIH [55]. Since the binding specificities of the anti-PD-1 antibodies from AIH patients and their ability to interfere with the PD-1/PD-L1 axis were not functionally validated, their pathogenic impact in breaking liver tolerance has yet to be determined. However, further evidence for the relevance of this axis derives from studies showing correlation of elevated soluble PD-1 (sPD-1) levels with activity [56, 57]. Secretion of sPD-1 which is generated via alternative splicing [58] might result in the competition of soluble and membrane-bound PD-1 on hepatic B and T cells for ligand interactions within the liver microarchitecture, thus interfering with the tolerogenic functions of PD-L1-expressing hepatocytes, liver sinusoidal epithelial cells (LSECs), hepatic stellate cells, dendritic cells (DCs), and Kupffer cells [59, 60].

### Hypergammaglobulinemia

In addition to autoantibodies, polyclonal hypergammaglobulinemia with a selective IgG elevation is another characteristic diagnostic hallmark of AIH [1, 3]. Elevated IgG levels in treated AIH patients mirror an ongoing inflammatory activity [61, 62], and normalization of serum IgG is an accepted treatment goal [37]. However, not all AIH patients have elevated IgG levels, and up to 15% of patients present with normal IgG levels when presenting with acute disease [37, 63]. This observation might be explained with varying baseline immunoglobulin levels due to genetic predisposition [64] which is in line with the observation that immunosuppression leads to a persistent decrease of IgG levels below normal thresholds in patients who present without IgG elevation in acute disease phases [63]. Recent findings have questioned the high selectivity of IgG elevation and suggest that hypergammaglobulinemia in AIH may also extend to IgA [63]. IgA antibodies are predominantly generated on mucosal surfaces [65], and increased serum levels might link alterations in gut microbiota and intestinal permeability to autoimmunity in general or AIH in particular [66–68].

### Pathological roles of hyper-IgG and autoantibodies

Despite their relevance for diagnosis and therapy monitoring, there is only limited evidence supporting a direct pathogenic role of immunoglobulins in AIH. Hepatocytes isolated from AIH patients are covered with immunoglobulin which may mediate antibody-dependent cellular cytotoxicity (ADCC) [69]. Immunoglobulin coating correlates with biopsy scores and portal but not parenchymal inflammatory activity [69]. In addition, anti-LKM1 can inhibit CYP2D6, which is expressed on hepatocyte plasma membrane, in vitro [70, 71]. However, serum transfer is not able to induce AIH in animal models [72], and fetal or neonatal hepatitis has not been reported in pregnant AIH patients [73, 74].

Other data suggest that autoantibodies and hypergammaglobulinemia might by a by-product reflecting loss of tolerance and/or an overshooting immune response. For example, IL-21 serum levels are elevated in AIH [75] and correlate with immunoglobulin levels [76]. Overexpression of IL-21 has been shown to be sufficient for autoantibody production and initiation of hypergammaglobulinemia in mouse models [77, 78]. IL-21 also triggers class-switching and the plasma cell differentiation program by regulating Blimp-1, Bcl-6, and Pax5 expression [77]. In addition, unpublished data from our group shows elevated IL-10 plasma levels clearly correlating with disease activity. The high plasma IL-10 levels could be seen as a regulatory response, since IL-10 is a dampener of excessive T cell activation while at the same time promoting immunoglobulin class switch recombination and secretion [79–82]. IL-10 has been described in diverse models of autoimmunity as main driver of autoantibody production (especially IgG) and secretion after initial activation by CD40L [83–85].

## Cellular functions of B cells in AIH

**Fig. 2:**
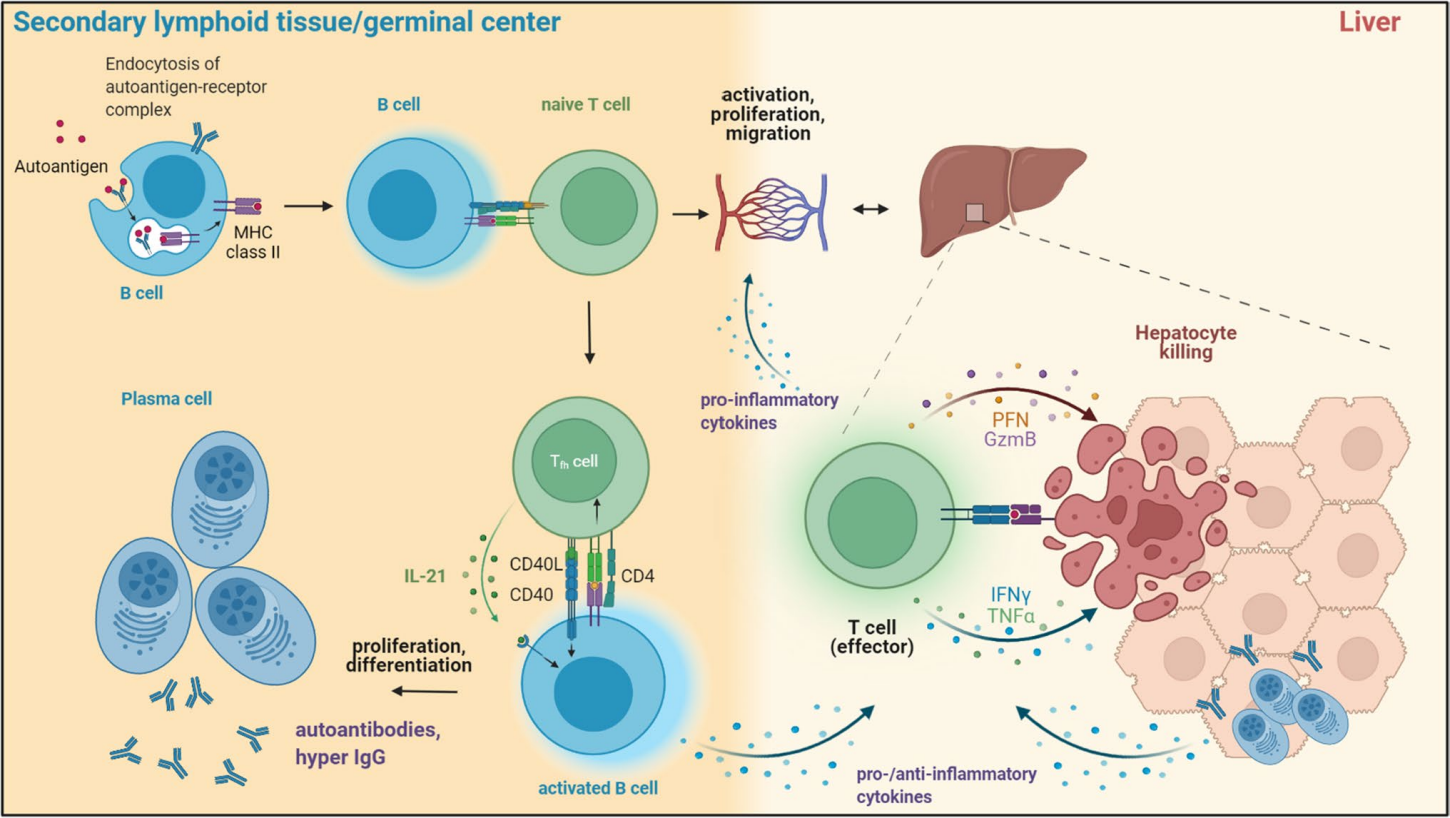
Potential roles of B cells in the pathogenesis of autoimmune hepatitis. B cells are professional antigen-presenting cells that can take up and present autoantigens processed by the endocytic pathway to naïve CD4^+^ T cells in the secondary lymphoid tissues. In addition, B cells can be primed by autoantigen activated T cells via interaction with T follicular helper (Tfh) cells in germinal center reactions (T cell help) and differentiate into (auto-)IgG secreting plasma cells and plasmablasts. Activated B and T cells migrate via the blood stream to target tissues and mediate tissue damage. They also secrete pro- and anti-inflammatory cytokines that potentially contribute to inflammation or counteract ongoing pathogenic autoimmune reactions

Immune repertoire sequencing can decode complex B and T cell architectures and identify immunogenetic imprints of infection [86], cancer [87], and immune-mediated diseases [88]. In our own recent work, immunosequencing of peripheral blood and liver-infiltrating B and T cell repertoires revealed a strong T cell receptor skewing unaffected by immunosuppression, while the B cell compartment was only marginally affected [89]. This is in line with flow cytometry data showing alterations in the composition of peripheral T cell subsets that persisted upon immunosuppresion, while B cells were not affected [90]. While these findings substantiate that AIH pathogenesis is not fueled by an antigen-driven B cell response, it does not exclude other essential regulatory B cell functions such as antigen presentation or cytokine and chemokine secretion exerted by distinct B cell subsets as important contributors of AIH pathogenesis. Indeed, the presentation of antigen to autoreactive CD4^+^ cells via B cells might provide a unifying link explaining both the HLA class II association and the various B cell abnormalities including enriched plasma cells in the liver infiltrate (Fig. 2). The regulatory B cell subset may also counteract T cell-mediated immune responses [91, 92].

### Antigen presentation

Genetic studies have shown that distinct polymorphisms in the human leukocyte antigen (HLA) region encoding the major histocompatibility complex (MHC) predispose to AIH [1, 3]. MHC class I (MHCI) and II (MHCII) are functionally similar heterodimeric proteins that present processed peptides to T cells [93]. While MHCI proteins are expressed by all nucleated cells and present proteasome-processed antigens of cytosolic and nuclear origin to CD8^+^ T cells, MHCII molecules are mainly expressed by professional antigen-presenting cells (APCs) like dendritic cells (DCs), macrohages, and B cells and present exogenous peptides processed by the endocytic pathway to CD4^+^ T cells [93]. Although the AIH-asociated polymorphisms vary between ethnic groups and geographical regions, they are located within the HLA-DR3 and HLA-DR4 loci encoding MHC class II molecules, thus suggesting a disease-driving role for CD4^+^ T cells [1, 3, 94]. Antigen presentation by B cells is considered a rate-limiting step for diverse autoimmune diseases by inducing activation of autoreactive T cells with the same specificity [95]. Autoreactive B cells that escape self-tolerance mechanisms in the bone marrow become anergic in the periphery and can populate secondary lymphoid tissues where they often locate at T-B cell borders allowing them to interact with antigen-specific T cells [95]. B cells can internalize antigen-bound BCRs via clathrin-mediated endocytosis, proteolytically process and load them on MHCII molecules for presentation [96] (Fig. 2). While peptide-MHCII-mediated T-B cell interactions are necessary for B cell maturation and class switiching (“T cell help”), MHCII antigen presentation by B cells can prime CD4^+^ T cells in the absence of other APCs and trigger the generation of memory and effector T cells in distinct settings [97, 98] (Fig. 2).

In an AIH mouse model, B cell depletion contributes to AIH by presenting antigens to CD4^+^ cells and caused a reduction of T follicular helper (Tfh) cell numbers [99]. Tfh cell differentiation is partly dependent on MHCII antigen presentation by B cells [100], and accumulation of Tfh cells has been shown to be necessary for autoimmunity [23]. Although their exact contribution to AIH pathogenesis is not understood, Tfh cell numbers are enriched in AIH patients [101] and mice models [99]. The role of Tfh cell-mediated T and B cell crosstalk for AIH pathogenesis is also substantiated by the finding that SLA-specific autoreactive T cells upregulate transcription programs associated with B cell help [102].

### Cytokine production

B cells can produce a broad array of pro- and anti-inflammatory cytokines and chemokines necessary for regulation of different aspects of immunity [103, 104] (Fig. 2). Examples for B cell-derived cytokines that contribute to T cell responses are, among others, IFNγ which promotes T_H_1 responses, IL‑2 which promotes T_H_2 memory responses, or TNF-α and CCL3 which regulate Th1 cell responses [104]. Rare subsets of B cells are reported as producers of IL-17 independent of IL-6 and IL-23 signaling and the RORγt transcriptiong factor [105]. In addition, IFN-γ inducible protein 10 (IP-10/CXCL10) which correlates with liver inflammation in AIH [106] is secreted by B cells (and hepatocytes) and mediates hepatic chemoattraction of T_H_1 and T_H_17 cells in AIH [107, 108].

Regulatory T cells (Tregs) are crucial for establishing tolerance, especially in the liver [109]. Most Tregs express heterodimeric IL-2 receptors (IL-2Rs) on their surface containing the high-affinitiy alpha chain (IL2RA/CD25) and are thus not highly responsive but also highly dependent on IL-2, a major factor for homeostasis as well as immunosuppressive and cytoprotective Treg function [110]. In a mouse model overexpressing the liver autoantigen FTCD, adoptive transfer of ex vivo IL-2 expanded CXCR3^+^ Tregs inhibited intrahepatic proliferation of autoreactive FTCD-specific B and T cells and restored peripheral tolerance [111]. AIH patients show decreased IL-2 serum levels and Tregs from AIH patients have been reported to be less responsive to IL-2 [112]. Low-dose IL-2 treatment in two patients with refractory AIH caused an increase in circulating Tregs and reduction of inflammatory liver damage [113].

A well-known driver of autoimmunity is IL-6 [114]. In multiple sclerosis models, B cell-derived IL-6 promotes the activation of pathogenic T_H_1 and T_H_17 cells, thereby driving the pathogenesis of this disease [115]. In AIH, IL-17 contributes to AIH pathogenesis by induction of hepatic IL-6 expression [116]. In line with this, several IL-6 polymorphisms are associated with AIH [117], and an effective second-line AIH therapeutic, 6-mercaptopurine, inhibits IL-6 production in B cells [118].

B cells, including plasma cells and plasmablasts, exert immunosuppressive functions by secreting distinct cytokines especially IL-10 and IL-35 [105, 119–121]. As mentioned above, our unpublished data shows elevated IL-10 plasma levels that correlated with marker normalization and disease remission. Here, IL-10 is most likely hypersecreted in active AIH to dampen high numbers of activated T cells and later downregulated when T cell activation gets more and more controlled. This provides a mechanistic basis for the finding that the immune system of AIH patients spontaneously attempts to counterregulate T cell autoreactivity in active disease [122] and also explains the observation of normal IgG levels in some patients with acute AIH [37]. Although IL-10 is mainly derived from distinct subsets of regulatory T cells and T_H_2 cells [81, 84], there is also a subset of regulatory B cells termed B10 solely characterized by intracellular production of IL-10 [123]. B10 cells inhibit inflammation in different model systems and humans but might also elicit proinflammatory functions [123]. However, it needs further investigation, if this subset has a relevant impact on B cell (de-)regulation in AIH. In addition, the immunosuppresive effects of IL-10 are partly mediated through upregulation of the membrane-associated E3 ubiquitin ligase MARCH1 which reduces the half-life of surface MHC-II complexes on antigen-presenting cells [124]. However, this is not exclusive for all B cell subets since IL-10 upregulates MHC-II complexes in follicular B cells [124–126].

IL-35 belongs to the IL-12 family and is a heterodimeric cytokine formed by p35 and EBI3 with immunosuppressive functions via increasing numbers of Tregs and regulatory B cells [127]. IL-35 fosters the generation of Tregs and inhibits CD4^+^ effector T cells like T_H_1 and T_H_17 cells [127, 128]. In AIH, the hepatic expression of the IL-35 subunits p35 and EBI3 is elevated and correlates with liver inflammation and fibrosis, the level of p35 also with age and serum levels of IgG and transaminases [129]. Notably, p35 is also a subunit of IL-12 (together with p40) and EBI3 of IL-27 (together with p28) [130]. Although p40 and p28 did not show equivalent correlations in immunohistochemical staining of liver tissue from AIH patients [129], higher expression of p35 and EBI3 might also hint towards production of IL-27 or IL-12; the latter of is also produced by B cells [131, 132]. Interestingly, transient hepatic overexpression of IL-12 in mice causes loss of tolerance to hepatocellular antigens leading to an AIH-like disease with hypergammaglobulinemia, autoantibodies, persistent immune cell infiltration of the liver, and hepatic fibrosis [133].

## Evidence for the pathogenic role of B cells in AIH from treatment studies

### Standard of care and impact on B cell function

Current therapies block pathogenic immune responses without reliably reestablishing immune tolerance [1, 3, 26, 134]. Standard of care for the induction of remission are the glucocorticoid derivatives prednisone or prednisolone, while azathioprine is given for the maintenance of remission with or without low levels of corticosteroids. In case of intolerance to azathioprine, its metabolite 6-mercaptopurine or mycophenolate mofetil (MMF) may be alternatives that alleviate adverse effects [135]. Functionally, these therapeutics represent nonspecific systemic immunosuppressants that have varying effects on immune cell types including B lineage cells.

Glucocorticoids bind the ubiquitously expressed cytosolic glucocorticoid receptor (GR) which acts as a ligand-inducible transcription factor after nuclear translocation [136–139]. In primary human B cells, GR bound prednisolone impairs BCR (downregulation of CR2/CD21, CD19, SYK, BTK, BLNK, and CD79B but not CD79A) and Toll-like receptor (TLR) 7 signaling, while immunosuppressive IL-10 and the marker for terminal plasma cell differentiation, PRDM1/Blimp-1, are upregulated [140]. Other reports showed that prednisolone inhibits proliferation, plasma cell differentiation, and IgG secretion in a dose-dependent manner and also reduces IL-10 and IL-21 cytokine levels, while low levels of prednisolone increase IgG secretion when added to peripheral blood mononuclear cells (PBMCs) [141–143]. In AIH patients, prednisolone therapy suppresses intrahepatic B and Treg cell proliferation and portal B and T cell densities [143]. Decline of B and Treg densities was proportional with similar Treg/B cell ratios before and under therapy [143]. In addition, portal CD79A^+^ B cell infiltrate density significantly correlated with serum IgG levels suggesting these cells as source of elevated IgG levels [143]. Given that mature B cells are more resistant as immature B cells towards long-term prednisolone administration although GR is expressed throughout all stages of B cell development [144, 145] and PRDM1/Blimp-1 is essential for maintenance of long-lived plasma cells (LLPCs) in the bone marrow [146], these findings might provide a mechanistic explanation for the observation that IgG normalizes upon glucocorticoid treatment, while autoantibody titers do not correlate with remission [37]. In this notion, glucocorticoid treatment would select an autoreactive B cell memory which persistently secretes autoantibodies, supports chronic inflammation, or might contribute to the fluctuating course of AIH including flares as also suggested for other autoimmune diseases [147].

Azathioprine is a pro-drug of 6-mercaptopurine that is converted by hypoxanthine–guanine phosphoribosyltransferase (HPRT1) to cytotoxic thioguanine nucleotides which are incorporated into newly synthesized nucleic acids and also reduce nucleotide synthesis by inhibiting enzymes of the purine metabolism [148]. The main immunosuppressive effect of azathioprine and its metabolites is attributed to blockade of DNA synthesis and proliferation of leukocytes by incorporation of cytotoxic purine analoga [148]. In addition, azathioprine-derived 6-mercaptopurine can also directly induce apoptosis in T cells by blocking the activity of the RAS-related C3 botulinum toxin substrate 1 (Rac1) GTPase [149]. It is reasonable to postulate the same mechanism for B cells which are highly sensitive to azathioprine [150] and dependent on Rac1 as mediator of BCR proliferation and survival signals [151]. Interestingly, low doses of azathioprine selectively reduce B cell numbers [152]. This property is used to effectively minimize the immunogenicity of anti-TNF antibodies and thus increase therapeutic efficacy [153, 154].

Mycophenolic acid (MPA) is the pharmacological active metabolite of MMF and is long known for its anti-inflammatory properties [148]. MPA reversibly inhibits inosine-5´-monophosphate dehydrogenase (IMPDH) and thus the formation of guanosine nucleotides with a high preference for T and B cells [155]. IMPDH has two isoforms, IMPDH1 and 2, from which IMPDH2 is more susceptible to MPA inhibition and also more abundant in lymphocytes [156]. MPA has a lower impact on B cell survival as compared to azathioprine, especially on antigen-naïve and resting memory B cells, but selectively inhibits B cell activation and plasma cell formation, while T cells appear not affected in SLE patients [157, 158]. MPA arrests B cells in the G0/G1 phase of the cell cycle and blocks immunoglobulin production from activated primary cells but not from terminally differentiated plasma cells expressing low levels of IMPDH2 [159]. Inhibition of immunoglobulin production of primary human B cells after CD40 ligation is independent of dose [160]. In addition, MPA reduces IL-6 production by B cells [118], which is linked to IL-17-driven AIH pathogenesis [116].

### B cell depletion in clinical AIH trials

Given the pivotal role of B cells for the development and outcome of many autoimmune diseases including AIH, therapeutic approaches targeting the B lineage emerge as promising therapeutic options. The majority of available treatment options are conceived as antibody-mediated B cell depletion therapies which either target the B cell-specific surface markers CD19 and CD20 or essential survival factors like the B cell activation factor (BAFF) as well as its homolog A proliferation-inducing ligand (APRIL) [161]. B cells express the CD20 molecule from the late pro-B cells to the development of memory cells, but lost during plasmablast/plasma cell differentiation [92]. Anti-CD20 antibody therapy with the monoclonal antibody rituximab has shown efficacy in the treatment of a range of autoimmune diseases such as rheumatoid arthritis, multiple sclerosis, pemphigus vulgaris, immune thrombocytopenia, or systemic lupus erythematosus by B lymphocyte depletion and decreased production of autoantibodies as reviewed [161–164]. However, since CD20 expression is lost on long-lived plasma cells, autoantibody production is not abrogated in all cases and might contribute to persistent inflammation or flares [147, 165].

In AIH, B cell depletion is so far only used as third-line therapy in small cohorts of difficult to treat patients showing promising results without safety concerns [166, 167]. A single center open label study of rituximab in 6 AIH patients and a retrospective multi-center cohort of 22 patients demonstrated significant improvements in serum IgG and liver transaminases sustained for up to 24 months after treatment and reported no significant adverse events [166, 167]. In paired liver biopsies of AIH before and after rituximab therapy, inflammation grade that correlated with CD4 regulatory T cells improved with treatment [166]. This suggests B cell depletion in AIH might work therapeutically through an indirect reduction in liver infiltrating CD4 T cells. However, prospective studies are yet to be obtained to validate the use of B cell depletion therapies in AIH. This is especially true for the long-term perspective of B cell depletion since anti-CD20 treatment of an AIH mouse model showed reduction in serum IgG but no histopathological normalization [168].

The success of rituximab led to the development of a second generation of humanized or full-humanized anti-CD20 (ocrelizumab, ofatumumab, ublituximab, obinutuzumab) and anti-CD19 (inebilizumab, obexelimab) antibodies [161] (Table 2). Currently, usage of next generation antibodies is evaluated in different autoimmune conditions; however, to our knowledge, no data is available about safety and efficiency in AIH [161].

**Table 2:** B cell targeting drugs

Name	Type/class	Target	Mechanism of action
**Direct targeting agents**			
Rituximab	Antibody	CD20	B cell depletion [218, 219]
Ocrelizumab	Antibody	CD20	B cell depletion [219]
Ofatumumab	Antibody	CD20	B cell depletion [219]
Ublituximab	Antibody	CD20	B cell depletion [220]
Obinutuzumab	Antibody	CD20	B cell depletion [221, 222]
Inebilizumab	Antibody	CD19	B cell depletion [223]
Obexelimab	Antibody	CD19	Supression of B cell activation by co-engaging with FcγRIIb [224, 225]
Ianalumab (VAY736)	Antibody	BAFF-R (TNFRSF13C)	BAFF blockade, depletion of BAFF-R^+^ B cells [177]
Belimumab	Antibody	BAFF (BlyS/TNFSF13B)	BAFF blockade [186], B cell depletion [226, 227]
**Indirect targeting agents**			
Abatacept	Fusion protein	CD80 (B7-1)/CD86 (B7-2)	Blocking CD28 [228]
Prednisone/prednisolone	Glucocorticoids	Nonspecific immunosuppressant	Downregulation of BCR signaling [140], cytokine regulation [142]
Azathioprine/6-mercaptopurine	Purine analogue	Nonspecific immunosuppressant	Cytotoxic purine antagonist that inhibits leukocyte proliferation [148]
Mycophenolate mofetil	biological compound	Nonspecific immunosuppressant	Inhibition of guanosine synthesis by blocking IMPDH [148, 155]

### Targeting of B cell regulating cytokines in AIH

BAFF and APRIL are crucial for survival and proliferation of B cells and plasma cells [169, 170]. These cytokines belong to the tumor necrosis factor family and are mainly provided by T cells and dendritic cells [171]. Both factors are known for their modulation of autoimmunity [161, 172, 173]. Self-tolerance can be achieved by an inactivation mechanism (anergy) which renders autoreactive B cells unresponsive to self-antigens. However, depending on the antigen, the anergic threshold necessary to stimulate B cells via their BCR can be overcome by BAFF-mediated signaling, thus activating autoreactive B cell clones [95]. Since BCR-coupled BAFF signaling defines a clone-specific threshold to rescue autoreactive B cells, low BAFF level maintain peripheral tolerance [174]. In AIH, BAFF levels are reported to correlate with liver inflammation [106, 175].

Ianalumab (VAY736) is an engineered, humanized, defucosylated, IgG1κ monoclonal antibody designed to block the BAFF receptor (BAFF-R/TNFRSF13C) and induce antibody-dependent cellular cytotoxicity (ADCC) of activated B cells. In primary Sjögren’s syndrome, treatment with ianalumab yielded improvements in salivary gland function, reduced tissue inflammation, sustained B cell depletion, and absence of major side effects [176, 177]. The anti-BAFF antibody belimumab has demonstrated promising results in different trials in SLE [178] and is therefore the first approved monoclonal antibody for treatment of SLE for patients intolerant or unresponsive for standard treatment [179]. First data in AIH show complete response in two AIH patients with refractory and advanced liver-related fibrosis who remained in remission while receiving low-dose corticosteroids. No adverse events related to belimumab and/or disease decompensation were observed [180]. Currently, the use of anti-BAFF receptor antibodies is evaluated in a randomized, placebo-controlled, double-blind dose range study in patients refractory or intolerant for standard treatment (NCT03217422).

### Targeting of B cell in their role as co-stimulators

Abatacept is a fusion protein comprising the extracellular domain of human CTLA-4. Therefore, it specifically inhibits the proliferation and activation of T cells by binding the surface markers CD80 and CD86 [181]. On B cells, abatacept binds CD80/CD86, thereby abrogating B cell-mediated co-stimulation of T cells. Studies in RA patients showed efficient decrease in symptoms, disease activity, and structural damage upon intravenous or subcutaneous administration [181]. Treatment of RA patients with abatacept showed CD80/86 downregulation on peripheral B cells. This was associated with decreased numbers of plasma cells as well as serum IgG levels [182]. Abatacept, which was successfully used in a RA case with adalimumab-induced hepatitis [183] and to treat graft-vs-host disease upon liver transplantation [184, 185], is currently investigated in treatment of recurrent or de novo AIH in liver transplanted patients (NCT04203875).

## Remarks and current research gaps/outlook

In summary, B cell-directed therapies such as blockade of BAFF and B cell-depletion have shown first evidence to be safe and efficient in treatment of AIH in patients unresponsive or intolerant to standard treatments. However, the experience of B cell-targeted therapies is limited, as only few cases or case series are reported, all of them were evaluated retrospectively. Therefore, special interest comes to the results of the first prospective phase 2 and 3 studies of ianalumab (NCT05124925, NCT05126277, NCT03656562, NCT02962895), one of which recently reported first data on safety and efficacy in patients with primary Sjögren’s syndrome [186]. Further, there is currently no data about the use of second generation of anti-CD20 antibodies in AIH as well as the use of antibodies targeting B cells in a broader spectrum of development, as it would be possible by the use of anti-CD19-targeted therapies. In addition, we lack information about targeting specifically plasma cells in AIH, which would be possible by use of a small molecule proteasome inhibitor bortezomib promoting plasma cell apoptosis. Bortezomib was shown to be efficient in various models of autoimmune diseases [187–189], and data from other autoimmune-mediated diseases provides evidence for efficiency in patients refractory to standard treatment [147, 190–200]. However, the potential side effects of, e.g., peripheral neuropathy, may limit its use. For evaluation of safety and efficiency, further studies are required.

The success of B cell-targeted therapies in AIH points out that B cells should not be considered innocent bystanders in AIH liver inflammation but rather substantial contributors to and mediators of pathogenic inflammatory processes. This can be facilitated either by being source of proinflammatory cytokines and (auto)antibodies, but also to provide help in sustaining the inflammatory state in AIH by supporting ongoing inflammation as B cells can also act as APC in secondary in chronic immune responses [97, 98, 201]. Irrespective of their substantial contribution to disease maintenance and progression, T cells still seem to play the pivotal role in disease onset in AIH [1–4, 202], and the use of B cell-targeted therapies as primary treatment in AIH remains questionable.

Since placebo-controlled trails of B cell-depletion therapies resulted in highly variable responses in other autoimmune diseases such as SLE [203], the multifaceted roles of B cells become clear. Also in RA B cells as producers of naturally arising antibodies (Nabs) are reported to be protective in the development of disease complications and might alter the disease burden [204]. Induction of regulatory B cells as potential mediators of regulatory functions with anti-inflammatory capacities is discussed as a novel treatment in autoimmunity. Regulatory B cells are reported to repopulate upon B cell-directed therapies, and their abundance are described to correlate with responsiveness to immunosuppressive treatment [162]. These hints to a more diverse role of B cells in autoimmune diseases, which to this point is only insufficiently understood in AIH.
